# Supplementary figures and images for: Analysis of Sphingolipids in Pediatric Patients with Cholelithiasis—A Preliminary Study
Source: J Clin Med. 2022 Sep 23;11(19):5613. doi: 10.3390/jcm11195613 (PMC9570855; doi:10.3390/jcm11195613)

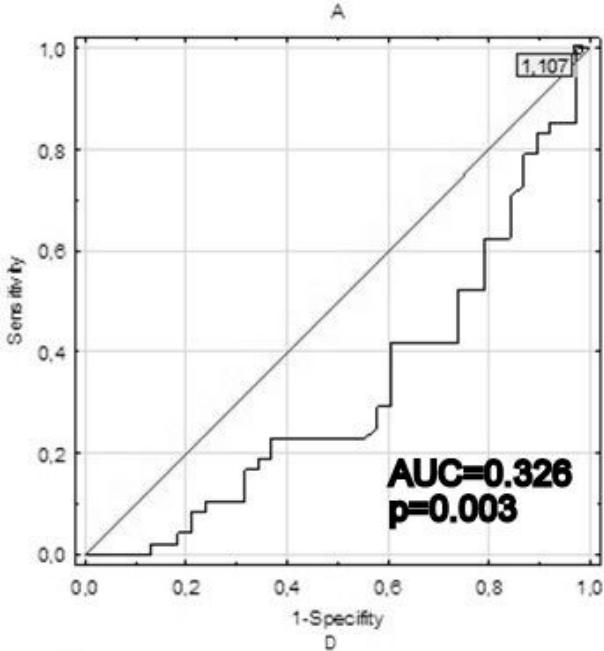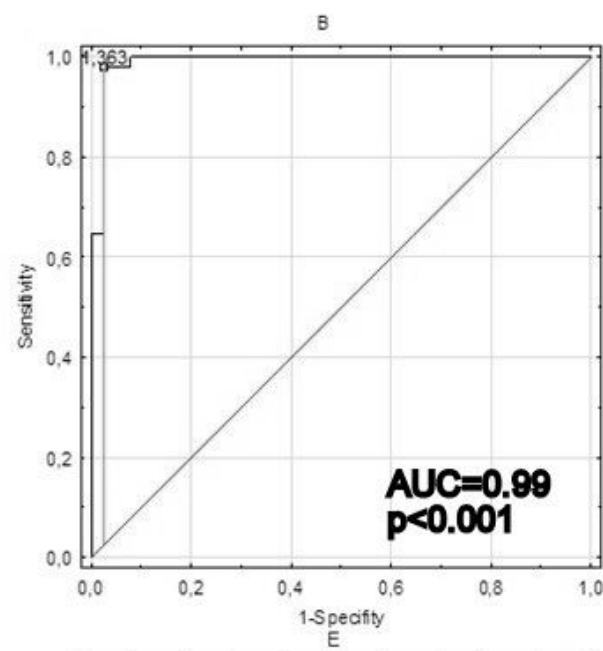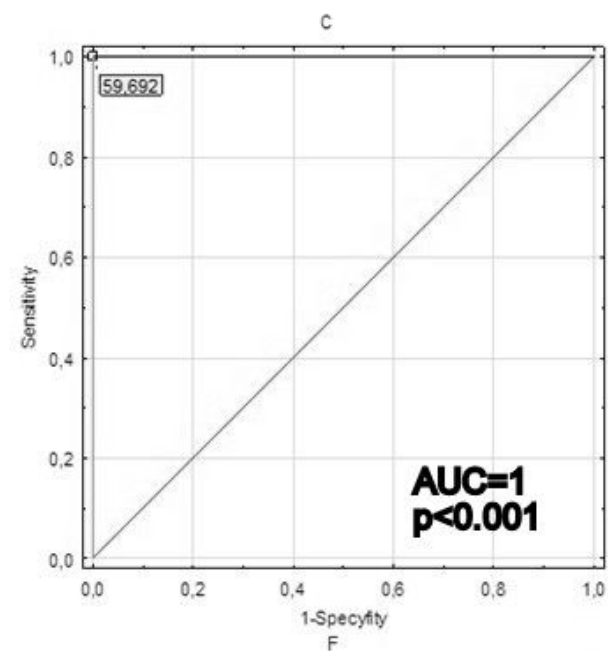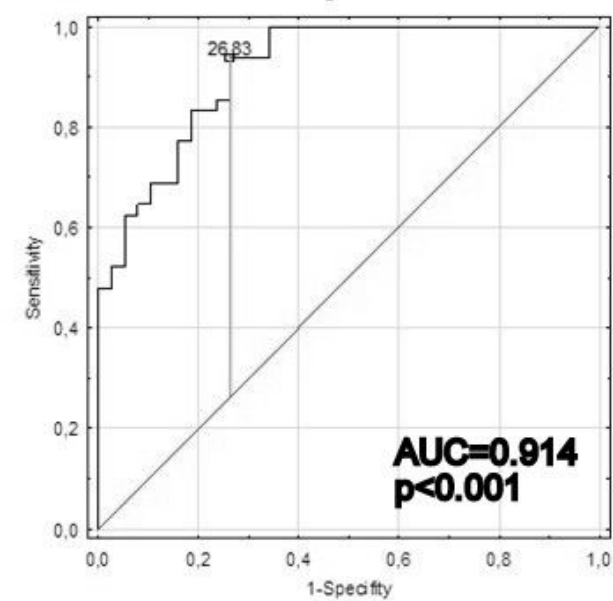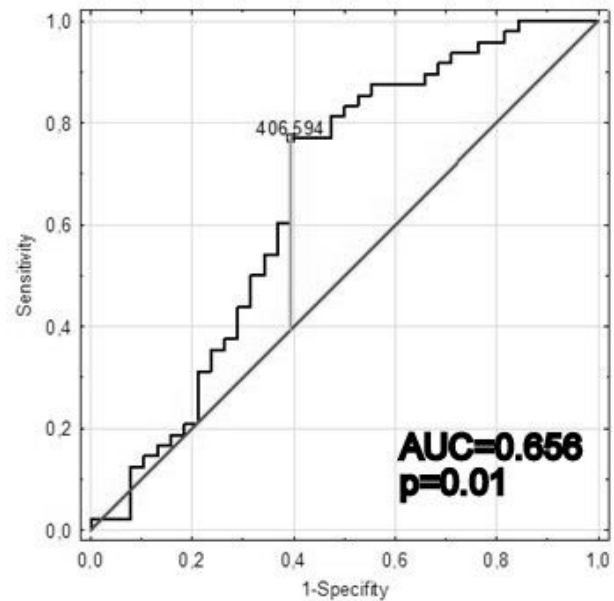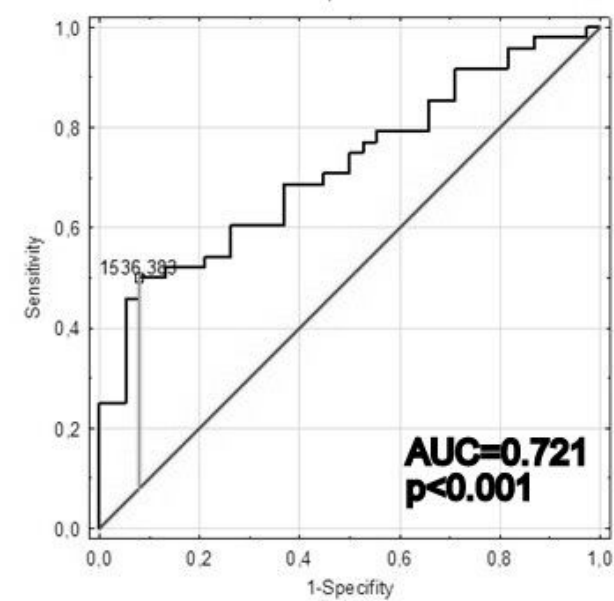

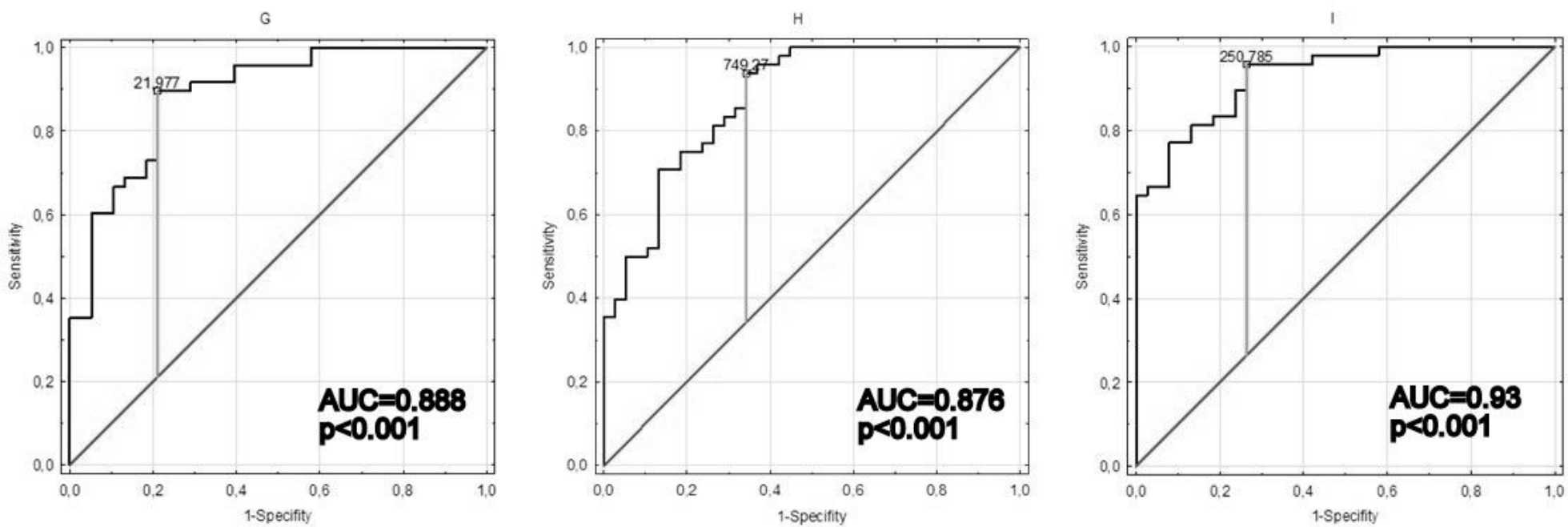

**Figure S1.** The ROC analysis.

Supplement: Supplementary file 1 [file jcm-11-05613-s001.zip › jcm-1832697-supplementary.pdf]
